# Biomimetic erythrocyte-based drug delivery systems for organ-targeted therapy

**DOI:** 10.3389/fbioe.2025.1663092

**Published:** 2025-09-09

**Authors:** Kehui Zhu, Yike Huang, Keying Li, Ke Zhang

**Affiliations:** ^1^ Department of Blood Transfusion, Sichuan Clinical Research Center for Cancer, Sichuan Cancer Hospital & Institute, Sichuan Cancer Center, University of Electronic Science and Technology of China, Chengdu, China; ^2^ Department of Laboratory Medicine, Clinical Laboratory Medicine Research Center, West China Hospital, Sichuan University, Sichuan Clinical Research Center for Laboratory Medicine, Chengdu, China

**Keywords:** red blood cells(RBCs), organ targeting, drug delivery system (DDS), cell therapeutic, cell drug delivery

## Abstract

Enhancing drug accumulation in target organs while minimizing adverse effects is critical for pharmacological therapies. Therefore, the development of advanced drug-targeting platforms is essential for clinical application. These systems must not only enable precise organ-specific targeting but also improve drug bioavailability and extend systemic circulation. In recent years, significant progress has been made in blood cell-inspired drug delivery strategies, with red blood cells-based (RBCs-based) platforms showing particular promise due to their inherent physiological advantages. Nevertheless, the development of organ-specific RBCs-mediated delivery systems remains challenging. We categorize and summarize various drug loading methods for existing RBCs, along with their advantages, disadvantages, and treated disease types. We then focus on describing various design strategies of RBCs-based delivery systems targeting specific organs and review their current applications. Additionally, we discuss current challenges and future perspectives regarding RBCs-based targeted delivery platforms.

## 1 Introduction

While some non-targeted drugs can be administered at clinically relevant doses without causing significant systemic toxicity, many chemotherapy agents lack tissue or organ specificity during systemic circulation and frequently induce systemic toxicity, damaging healthy tissues (Adepu and Ramakrishna, 2021; Chen et al., 2020; Nie et al., 2023). To address this limitation, extensive research over the past decades has focused on developing carrier systems that enhance safety, efficacy, and targeting specificity. An ideal drug carrier should meet the following criteria (Adepu and Ramakrishna, 2021; Beach et al., 2024; Li et al., 2022; Liu W et al., 2024; Nie et al., 2023): 1) prolong the *in vivo* circulatory half-life of drugs while avoiding rapid immune clearance; 2) improve targeting precision to the therapeutic site; and 3) minimize toxicity to healthy tissues and organs.

Targeted nanocarrier delivery systems represent a groundbreaking advancement in precision medicine, offering innovative approaches for biomedical diagnosis and therapy. Notably, nanoparticles (NPs) with specific targeting capabilities demonstrate remarkable advantages by overcoming physiological drug delivery barriers, enabling selective drug accumulation in target tissues and cells while significantly prolonging systemic circulation time. This technological platform provides novel solutions to circumvent the limitations of conventional drug delivery systems.

Despite their widespread use, NPs have a major limitation: most administered NPs accumulate in reticuloendothelial system (RES)–rich tissues, particularly the liver and spleen, where they are rapidly cleared by macrophages, leading to a short half-life and low bioavailability (Cheng et al., 2020; Zahednezhad et al., 2019; Zhang et al., 2016). A recent study (He et al., 2024) reported that typically less than 1% of administered NPs reach target organs, with the vast majority sequestered in the liver, underscoring that current NP systems remain suboptimal in targeting specificity, pharmacokinetics, and biocompatibility. To enhance NPs circulation and evade clearance by the RES, polyethylene glycol (PEG) modification is commonly employed (Dabagh et al., 2023; Mahmood et al., 2023). However, they carry risks such as drug leakage and the accelerated blood clearance (ABC) phenomenon, which may result in liver and kidney toxicity (Lin et al., 2023; Saadati et al., 2013). Repeated administration of such exogenous materials can activate the host immune system, leading to suboptimal clinical outcomes in terms of pharmacokinetics, biocompatibility, and therapeutic efficacy (Gautam et al., 2023; Saraiva et al., 2016).

To overcome these limitations, researchers have begun to focus on “biomimetic carriers” as a promising drug delivery platform. Since blood is the primary medium for intravascular drug transport, endogenous blood cells may represent ideal drug delivery vehicles (Hadi Barhaghtalab et al., 2024; Jia et al., 2025). Among various drug carriers, red blood cells (RBCs) are the most abundant, comprising over 99% of all blood cells (Choi et al., 2023; Huynh et al., 2023). Lacking nuclei and organelles, RBCs possess a large surface area (∼160 μm^2^) and a long lifespan of 80–120 days (Brenner et al., 2021; Muzykantov, 2010) in contrast to the much shorter lifespans of platelets (Schlesinger, 2018), leukocytes (Puidokas et al., 2019), and macrophages (Wynn and Vannella, 2016) (7–21 days). RBCs also exhibit remarkable deformability, enabling them to traverse capillaries smaller than their own diameter (Ebrahimi and Bagchi, 2022; Reale et al., 2023). Additionally, Surface-enriched immunomodulatory proteins (notably CD47 and phosphatidylserine) mediate macrophage evasion through “don’t-eat-me” signaling, significantly enhancing the circulatory persistence of nanotherapeutics (Velliquette et al., 2019; Villa et al., 2016; Wiewiora et al., 2017). These properties make RBCs an ideal natural carrier for vascular drug delivery.

**FIGURE 1:**
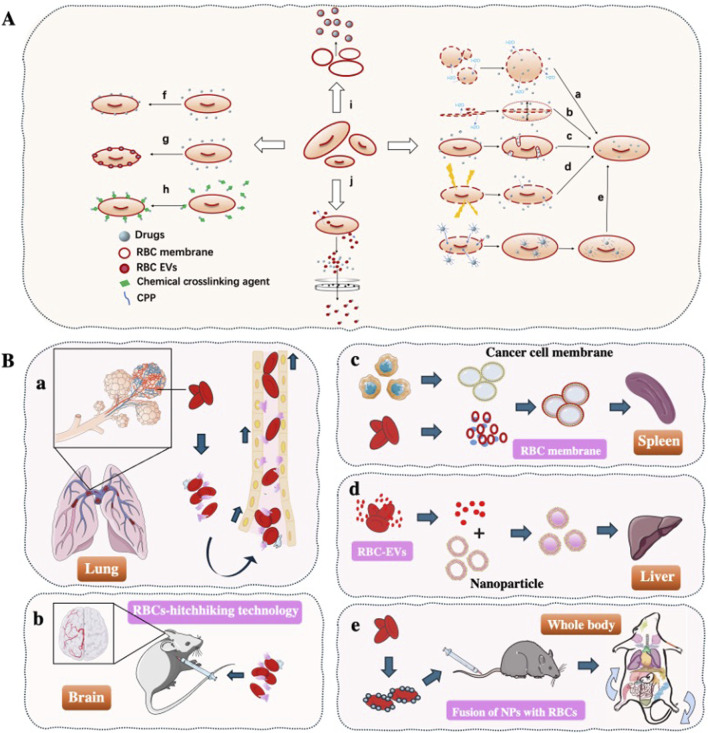
Overview of Red Blood Cells as Drug Delivery Vehicles **(A)** The drug loading method of RBCs drug delivery system. (a-b) Osmotic lysis and resealing; (c) Induced endocytosis; (d) Electroporation; (e) Cell-penetrating peptides; (f) RBCs-hitchhiking; (g) membrane fusion techniques; (h) Chemical conjugation; (i) RBCs membrane-coated nanoparticles; (j) RBCs-EVs for drug encapsulation. **(B)** Organ targeting strategies using RBCs. (a) By modifying the injection site of drugs, RBCs-hitchhiking technology enables targeted delivery of NPs to either lungs or brain; (c) Fusion of tumor cell membranes with RBCs membranes induces antigen responses *in vivo*, promoting tumor antigens delivery to the spleen to enhance cancer immunotherapy; (d) The natural liver-targeting capability of RBCs-EVs enables drug delivery to the liver; (e) Fusion of NPs with RBCs prolongs systemic circulation time while reducing non-target organ uptake, achieving sustained whole-body drug delivery.

The use of RBCs for drug delivery dates back to 1950s, when (Gardos, (1954) first demonstrated RBCs-mediated delivery of adenosine triphosphate (ATP) in experiments performed in 1953 and published in 1954, marking the inception of erythrocyte-based drug carriers. Although emerging blood-borne pathogens (including HIV and *Treponema pallidum*) have constrained the clinical translation of RBCs-based drug delivery systems, the remarkable circulatory persistence of RBCs makes their development as therapeutic carriers highly promising. This approach holds significant potential for treating diverse diseases (Belov et al., 2023; Della Pelle and Kostevsek, 2021). Over the decades, various RBCs-loading strategies have been developed, including (Figure 1A): (a-b) osmotic lysis and resealing (Gutierrez-Millan et al., 2023); (c) induced endocytosis (Harisa Gel et al., 2011); (d) electroporation (Peng et al., 2023); (e) cell-penetrating peptides (CPPs) (He et al., 2014); (f) RBCs-hitchhiking (RH) (Nguyen et al., 2023); (g) membrane fusion techniques (da Silveira Cavalcante et al., 2017; Stoll et al., 2011); (h) chemical conjugation (Li et al., 2024); (i) RBCs membrane (RBCM) coated nanoparticles (Hayashi et al., 2018a; He et al., 2023), and (j) RBCs-derived extracellular vesicles (RBCs-EVs) for drug encapsulation (Liu et al., 2022; Valkov et al., 2021). Numerous studies (Cheng and Wang, 2024; Krivic et al., 2022; Xia et al., 2019) have demonstrated that RBCs can serve as either direct or indirect drug carriers for targeted delivery to various organs including lungs, brain, liver, and spleen, depending on therapeutic requirements. Importantly, for specific disease treatments (e.g., lymphoma, leukemia), RBCs-based delivery systems significantly reduce drug uptake by non-target organs while simultaneously achieving prolonged systemic circulation and enhanced therapeutic efficacy (Figure 1B). This review summarizes the organ-targeting capabilities of current RBCs-based delivery systems and highlights their advantages over synthetic carriers for intravascular transport. Moreover, we discuss how various processing techniques influence RBCs tropism toward different organs, underscoring their potential for treating a wide range of diseases.

### 1.1 Current RBCs-based drug delivery systems

Current RBCs-based drug delivery systems can be primarily categorized into three types: intact RBCs, RBCs-EVs, and RBCM. In this section, we focus on reviewing existing RBCs-based drug delivery systems, discussing their respective advantages, limitations, and future development prospects, with representative cases summarized in Table 1.

**TABLE 1:** Overview of current RBCs-based drug delivery systems.

Carrier	Drug localization	Loading method	Cargo	Disease	References
RBCs	Intracellular	Hypotonic	L-asparaginase Dexamethasone sodium Glucocorticoids Dexamethasone	Pancreatic carcinoma Breast cancer Telangiectasis Targeting macrophages Lymphoma leukemiaetc.	Agrawal et al. (2013) Mambrini et al. (2017) Gutierrez-Millan et al. (2023) Zhang et al. (2018)
		Hypertonic	Morphine	Postoperative analgesia	Carrasco-Sánchez et al. (2014)
		Induced endocytosis	Primaquine Pravastatin	Malaria Increase the drug circulation time	Harisa Gel et al. (2011)
		Electroporation	Interleukin-2 Doxorubicin Indocyanine green	Colon cancer Tumor immunotherapy Anti-inflammatory	Peng et al. (2023) Lucas et al. (2019)
		Cell-penetrating peptides	L-asparaginase	Lymphoma leukemia	He et al. (2014)
	Membrane surface	Chemical conjugation	Doxorubicin Antibody-lipid conjugates	Cancer therapy Targeting cancer cells	Li et al. (2024) Li et al. (2020) Ji et al. (2020)
		RBCs-hitchhiking	Polystyrene NPs Doxorubicin-NPs CLCX-10 Paclitaxel	Melanoma Pulmonary metastasis Transplantation tumor	Zelepukin et al. (2019) Brenner et al. (2021) Zheng et al. (2022) Sun et al. (2024)
		Membrane fusion	Trehalose Paclitaxel	Protect cell Increase the drug circulation time	Stoll et al. (2011) Zhu et al. (2023)
RBCs-EVs	-	Fusion	Doxorubicin RNA drugs	Hepatoma Safe Delivery of RNA drugs	Hou et al. (2022) Usman et al. (2018) Sangha et al. (2023)
RBCM	-	Fusion	Docetaxel Probucol Multifunctional-NPs	Subcutaneous tumor Orthotropic glioma Atherosclerosis Hepatitis B virus Enhancing cancer immunotherapy	Liang et al. (2022) Yang et al. (2024) Xu et al. (2023) Chen et al. (2023)

#### 1.1.1 RBCs-based drug delivery system

Drug loading into viable RBCs primarily employs two distinct strategies: intracellular encapsulation within the RBCs and surface conjugation to the RBCs membrane.

##### 1.1.1.1 Osmotic methods

Numerous studies have employed osmotic pressure modulation, induced endocytosis, and electroporation for RBCs drug loading.

Osmotic methods involve exposing RBCs to hypertonic or hypotonic conditions to create transient membrane pores for drug encapsulation (Figure 1Aa-b) (Chen et al., 2019). Magnani at EryDel developed the “Red Cell Loader” system using this approach, successfully loading dexamethasone-21-phosphate for treating COPD and pulmonary fibrosis (Mambrini et al., 2017). Bourgeaux et al. at Erytech similarly created the “ERY-caps” device to encapsulate L-asparaginase for acute lymphoblastic leukemia (ALL) treatment (Mambrini et al., 2017). Clinical studies have also demonstrated (Carrasco-Sánchez et al., 2014) successful morphine loading via hypertonic methods. Despite advantages including operational simplicity, rapid processing, and scalability, osmotic techniques cause significant membrane damage, leading to irreversible RBCs injury and accelerated RES clearance *in vivo* (Gutierrez-Millan et al., 2023).

##### 1.1.1.2 Induced endocytosis

Ginn et al. first reported (Ginn et al., 1969) that primaquine could induce RBCs membrane endocytosis, facilitating passive drug incorporation into the inner phospholipid bilayer (Figure 1Ac). Building on this, N.K. Jain et al. demonstrated (Talwar and Jain, 1992) that glutaraldehyde-treated RBCs loaded with primaquine achieved liver and spleen targeting. Subsequent studies achieved 94% encapsulation efficiency for pravastatin using this method. In 2011, systematic optimization of time, temperature, and concentration enabled RBCs-based sustained pravastatin delivery (Alanazi et al., 2011). However, this approach is limited to amphiphilic drugs and suffers from instability, restricting its therapeutic potential.

##### 1.1.1.3 Electroporation

Electroporation induces transient membrane permeability by creating nanopores via electrical pulses (Figure 1Ad). Mitchell achieved 5%–7.5% encapsulation efficiency for recombinant human interleukin-2 using this method, which has since been adapted for diverse small/large molecules in human RBCs(Mitchell et al., 1990). Notably, Peng et al. co-loaded indocyanine green (ICG) and doxorubicin (DOX), demonstrating (Peng et al., 2023) tunable tumor microenvironment modulation and synergistic antitumor efficacy *in vivo*. This method enables uniform drug distribution within RBCs. However, it requires specialized instrumentation and complex procedures, while yielding suboptimal encapsulation efficiency. Importantly, Tsong et al. observed (Tsong, 1991) post-electroporation RBCs lysis due to colloidal osmotic imbalance-induced ion dysregulation, leading to rapid cell death.

##### 1.1.1.4 CPPs

As evidenced above, most existing RBCs drug-loading methods inevitably cause RBCs damage. To address this limitation, CPPs have emerged as a promising alternative that reduce pore formation and mitigate hemolysis (Figure 1Ae). He et al. demonstrated (He et al., 2014) the efficacy of CPP-mediated delivery by successfully loading L-asparaginase into RBCs, which nearly doubled the enzyme’s blood half-life and significantly improved overall survival rates in preclinical models.

The exceptionally large surface area of individual RBCs presents a significant advantage for drug delivery. Current approaches for surface loading primarily include Chemical conjugation, RBCs-hitchhiking, membrane fusion.

##### 1.1.1.5 RBCs-hitchhiking technology (RH technology)

The RH technology involves attaching NPs to RBCs surfaces via electrostatic, van der Waals, or hydrophobic interactions (Figure 1Af). Following intravenous administration, RBCs-NPs complexes accumulate in capillary-rich tissues through mechanical squeezing forces, significantly reducing hepatic/splenic uptake while enhancing pulmonary accumulation (Zelepukin et al., 2019). This strategy enables effective lung-targeted delivery while minimizing systemic adverse effects. However, RH technology faces two major limitations (Sun et al., 2024; Zhao et al., 2021): 1) NPs dissociation due to weak RBCs-NPs interactions; 2) Low targeting efficiency (approximately 3% of initial dose reaches lungs), with most detached NPs being cleared by the RES.

##### 1.1.1.6 Liposome and RBCs membrane fusion

Another promising drug delivery approach involves fusing RBCs with drug-loaded liposomes, which reportedly outperforms hypotonic methods in encapsulation efficiency, phosphatidylserine (PS) exposure, and RBCs deformability (Figure 1Ag). Studies demonstrate (Stoll et al., 2011; da Silveira Cavalcante et al., 2018) that fusion efficiency depends critically on liposome membrane fluidity, lipid-to-cell ratio, incubation time/temperature, and solution composition, with lipid components modulating uptake mechanisms. Holovati et al. revealed (Holovati et al., 2008a) temperature-dependent interactions: at 37 °C, liposomes primarily fuse with or undergo endocytosis by RBCs membranes, while at lower temperatures they mainly adsorb to the surface. This method preserves native RBCs characteristics while significantly prolonging drug half-life for enhanced therapeutic efficacy (Zhu et al., 2023). However, challenges include fusion-induced hemolysis, numerous optimization parameters requiring substantial preclinical optimization, and difficulties in large-scale production of drug-loaded RBCs. These hurdles currently impede industrial-scale application.

##### 1.1.1.7 Chemical conjugation

Current studies have successfully conjugated protein molecules to RBCs membranes using non-specific chemical crosslinkers such as glutaraldehyde, which links amine groups on both the drug and RBCs surface (Li et al., 2024) (Figure 1Ah). The conjugated RBCs release therapeutic payloads via circulatory hydrolysis. Studies (Ji et al., 2020) have conjugated up to 100,000 glucose molecules onto RBCs surfaces, enabling prolonged systemic circulation *in vivo*. However, excessive chemical modification may compromise RBCs biocompatibility and deformability, potentially inhibiting CD47 (“do not eat me” signal) or damaging other protective membrane proteins, while also inducing reactive oxygen species (ROS) generation (Li et al., 2020).

#### 1.1.2 RBCM based drug delivery system

This approach confers NPs with prolonged circulation and sustained drug release by coating them with RBCM (Figure 1Ai). Studies have confirmed (Hou et al., 2022) the successful encapsulation of Fe_3_O_4_-NPs, with TEM revealing correct membrane orientation (outer leaflet outward) that minimizes immune clearance. Wibroe et al. demonstrated (Wibroe et al., 2017) that porcine RBCM-coated NPs attenuate cardiopulmonary adverse effects by delaying macrophage recognition, thereby extending systemic circulation. This biohybrid system exhibits considerable therapeutic potential.

#### 1.1.3 RBCs-EVs based drug delivery system

Extracellular vesicles (EVs) (Di et al., 2023; Lehmann et al., 2023; Meng et al., 2020) are endogenous nanocarriers secreted by cells, showing promising potential as delivery platforms due to their excellent biocompatibility, tissue tropism, and minimal cytotoxicity and immunogenicity. RBC-EVs are nanosized membrane vesicles (50–200 nm) actively released by RBCs under physiological conditions or external stimuli such as hypotonic treatment and electroporation (Figure 1Aj). Emerging evidence supports their significant potential in gene therapy, cancer vaccines, and organ-specific drug delivery. Unlike artificially extracted RBCM, RBC-EVs are naturally secreted vesicles with superior biocompatibility. Table 2 details the characteristics and differences between these two RBC-derived carrier systems (Ma et al., 2023; Wannez et al., 2019; Xia et al., 2019; Yu et al., 2023). In recent years, RBCs-EVs have emerged as an ideal drug delivery platform due to their low immunogenicity and prolonged circulation time *in vivo*. Studies demonstrate (Hou et al., 2022; Sangha et al., 2023) that RBCs-EVs adsorbed onto NPs effectively retain native membrane proteins while maintaining excellent stability. RBCs-EVs coated silica-NPs demonsrate high drug-loading capacity, minimal leakage, and enhanced stability in breast cancer mouse models (Sangha et al., 2023). In 2020, Zhang et al. electroporated DOX or sorafenib into RBCs-EVs, which achieved natural liver targeting without additional modification, significantly suppressing orthotopic liver tumor growth in mice (Xiu et al., 2022; Zhang et al., 2020). Importantly, RBCs-EVs-based delivery has demonstrated excellent biosafety, highlighting its potential as a novel therapeutic for liver diseases. However, challenges remain in large-scale production and quality control, and clinical translation remains unachieved (Hadi Barhaghtalab et al., 2024).

**TABLE 2:** The main differences between RBC-EVs and RBCM.

	RBC-EVs	RBCM
Source	Native vesicles retaining select membrane proteins and cytoplasmic constituents	Artificially extracted RBCM recoated on NPs
Preparation	Ultracentrifugation, hypotonic stimulation	Membrane extraction followed by fusion with NPs (e.g., sonication, extrusion)
Drug Loading	Endogenous loading (passive/active drug entrapment)	Exogenous loading (pre-encapsulation in NPs)
Size Range	50–200 nm (closer to natural exosomes)	80–200 nm (core-dependent)
Immune Evasion	High (retains CD47″do not eat me” signals)	High (but may lose some membrane proteins during processing)
Targeting	Relies on natural membrane proteins, and RBCs-EVs exhibit intrinsic liver-targeting properties	Engineerable, and RBCM exhibits homing effects to the spleen
Clinical Potential	Excellent biocompatibility (fully endogenous)	Scalable production but requires strict quality control

### 1.2 RBCs-based drug delivery systems for organ-targeted therapy

Based on the aforementioned advantages, RBCs’ inherent biological properties provide an excellent biological and structural foundation for drug carrier development. This section focuses on targeted organ delivery using RBCs-based drug delivery systems.

#### 1.2.1 Lung

**FIGURE 2:**
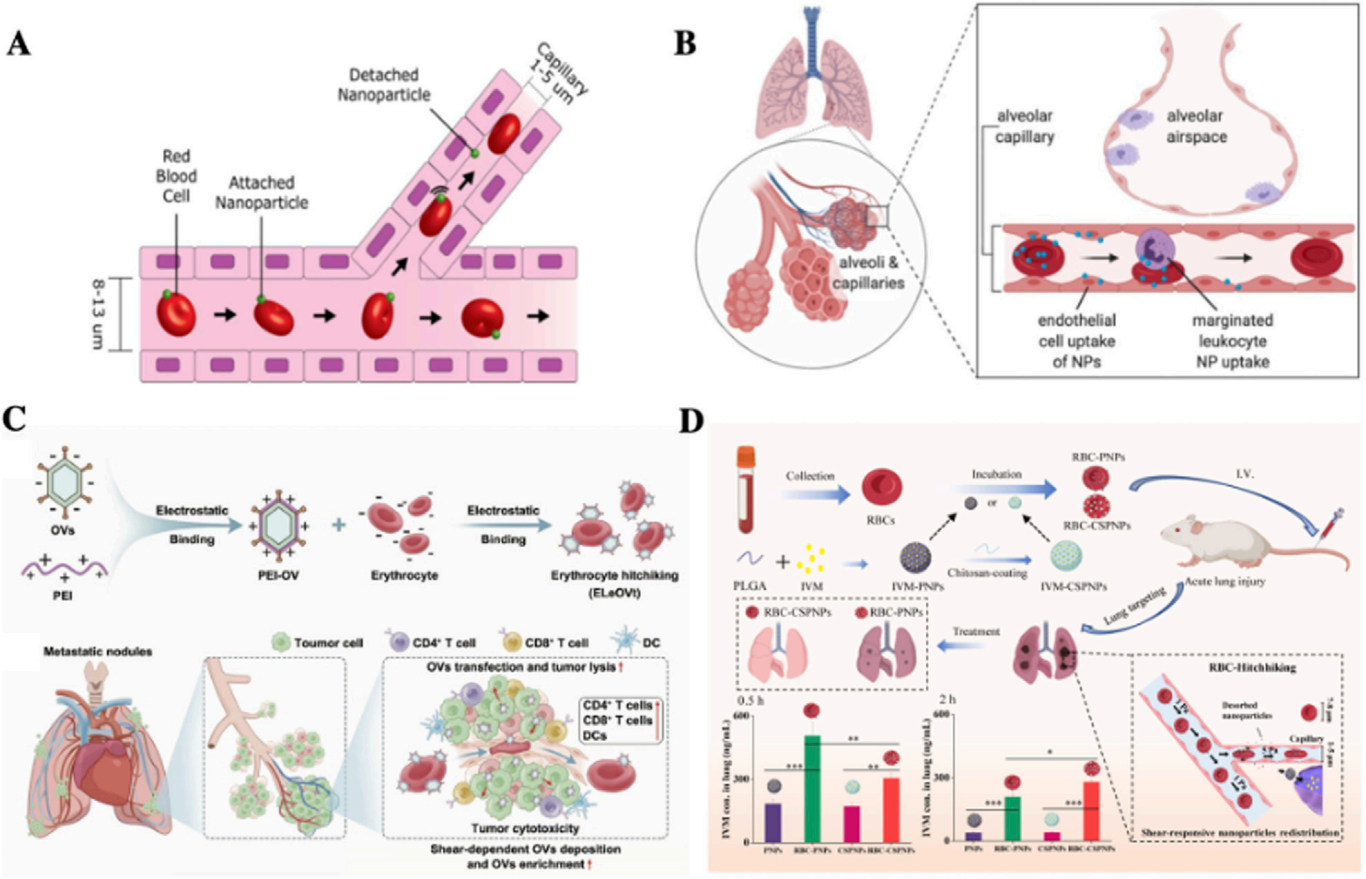
Applications of RBCs as carriers for pulmonary drug delivery. **(A)** Schematic illustration of the RH technique (Anselmo et al., 2013); **(B)** In the lung, the best studied organ of uptake in RH, RH transfers NPs to endothelial cells (Brenner et al., 2021); **(C)** Conjugate of PEI to RBC surfaces via RH prolongs drug half-life, reduces hepatotoxicity, and markedly inhibits pulmonary tumor metastasis (Liu M et al., 2024); **(D)** ivermectin (IVM)-loaded NPs with various materials, sizes, and surface modifications were non-covalently adsorbed onto RBCs to form RBC-NP complexes, enabling targeted delivery to inflamed lung tissue for inflammation treatment (Zheng et al., 2022).

The primary physiological function of RBCs is oxygen transport (Amreddy et al., 2018). As all circulating RBCs pass through the lungs for oxygenation, drug-loaded RBCs represent a promising strategy for enhancing drug delivery to pulmonary lesions (Hima et al., 2023). In 2013, Anselmo et al. (2013) demonstrated that when polylactic-co-glycolic acid (PLGA) NPs detached from RBCs, they were taken up by pulmonary capillary endothelial cells, thereby markedly reducing clearance by the RES. Their findings revealed that RBCs-NPs exhibited prolonged systemic circulation and enhanced pulmonary drug accumulation in comparison with free NPs. This led to the development of RBCs-hitchhiking (RH) technology, whereby NPs are attached to the surface of RBCs via electrostatic interactions, van der Waals forces, or hydrophobic interactions (Figure 2A). During systemic circulation, NP-loaded RBCs deformed to pass through narrow capillaries, resulting in mechanical detachment of NPs, which subsequently accumulate in capillary-rich tissues or organs. Thus, when RBCs reach the lungs, the first capillary bed encountered after intravenous injection, drug-carrying NPs are deposited into the pulmonary circulation (Figure 2B).

Due to the lungs’ dense capillary network, nearly all primary malignant tumors have the potential to metastasize to the pulmonary system (Xie et al., 2021). The advent of RH technology has therefore opened new avenues for RBCs-based drug delivery systems targeting lung cancer. Zelepukin et al. (2019) reported that approximately 100 nm NPs loaded onto RBCs via RH technology achieved pulmonary accumulation of up to 40% of the injected dose. This strategy effectively suppressed melanoma lung metastasis, highlighting its potential in treating metastatic cancer. In recent years, RH technology has undergone further refinement. For instance, Zhao et al. (2021) developed NPs encapsulating the CXCL10, which recruits effector immune cells. By combining CXCL10-NPs with RH technology, they achieved enhanced pulmonary targeting and concurrent systemic immune activation, leading to reduced pulmonary tumor metastasis. In 2023, Huynh et al. (2023). Introduced a lung-targeted delivery system based on RH technology using multigrain iron oxide nanostructures (MICs). This system was specifically designed to suppress lung cancer metastasis and recurrence while minimizing immune cell infiltration and hepatorenal toxicity associated with poor pulmonary targeting. Experimental results demonstrated that over 65% of the delivered drug localized in tumor tissues rather than in normal lung tissues, demonstrating an approximate 20-fold enhancement in lung-targeting efficiency compared to conventional delivery methods. In 2024, (Liu M et al., 2024). Further improved RH technology by electrostatically loading positively charged polyethyleneimine (PEI) onto RBCs surfaces (Figure 2C). This approach augmented the efficacy of oncolytic virotherapy by utilizing RBCs surface markers (e.g., CD47) to evade immune surveillance, thereby prolonging circulation time and reducing hepatotoxicity. The modified system exhibited significant efficacy in suppressing pulmonary tumor metastasis.

Beyond cancer therapy, the intrinsic lung-targeting properties of RH technology have demonstrated therapeutic potential across a range of pulmonary diseases. A prominent example is COVID-19, which emerged in 2019 and is characterized by pulmonary hemorrhage, epithelial injury, and inflammatory-induced lung damage (Hu et al., 2021; Stasi et al., 2020). While anti-inflammatory agents may exacerbate acute lung injury, glucocorticoids, with their dual anti-inflammatory and immunomodulatory properties, have been widely employed (de Lemos Neto et al., 2021; Ricciotti et al., 2021). However, high-dose glucocorticoid therapy is often associated with severe side effects and steroid dependence (Akbas and Akbas, 2021; Alexaki and Henneicke, 2021; Yang and Yu, 2021). A 2022 study (Zheng et al., 2022) addressed these challenges by loading glucocorticoid-NPs onto RBCs surfaces, significantly extending systemic circulation and reducing hepatic and splenic accumulation (Figure 2D). The findings showed that RH technology markedly increased pulmonary drug accumulation, thereby offering an effective platform for targeted therapy in pulmonary conditions. In the following years, RH based strategies were extensively applied in viral pneumonia research. Their superior lung-targeting capabilities successfully addressed limitations in conventional treatment using ivermectin (IVM) and simvastatin (SIM) for acute respiratory distress syndrome (ARDS), including poor bioavailability, short half-life, and suboptimal tissue distribution. Collectively, these advances underscore the unique suitability of RBCs as carriers for pulmonary drug delivery (Zheng et al., 2022).

#### 1.2.2 Brain

**FIGURE 3:**
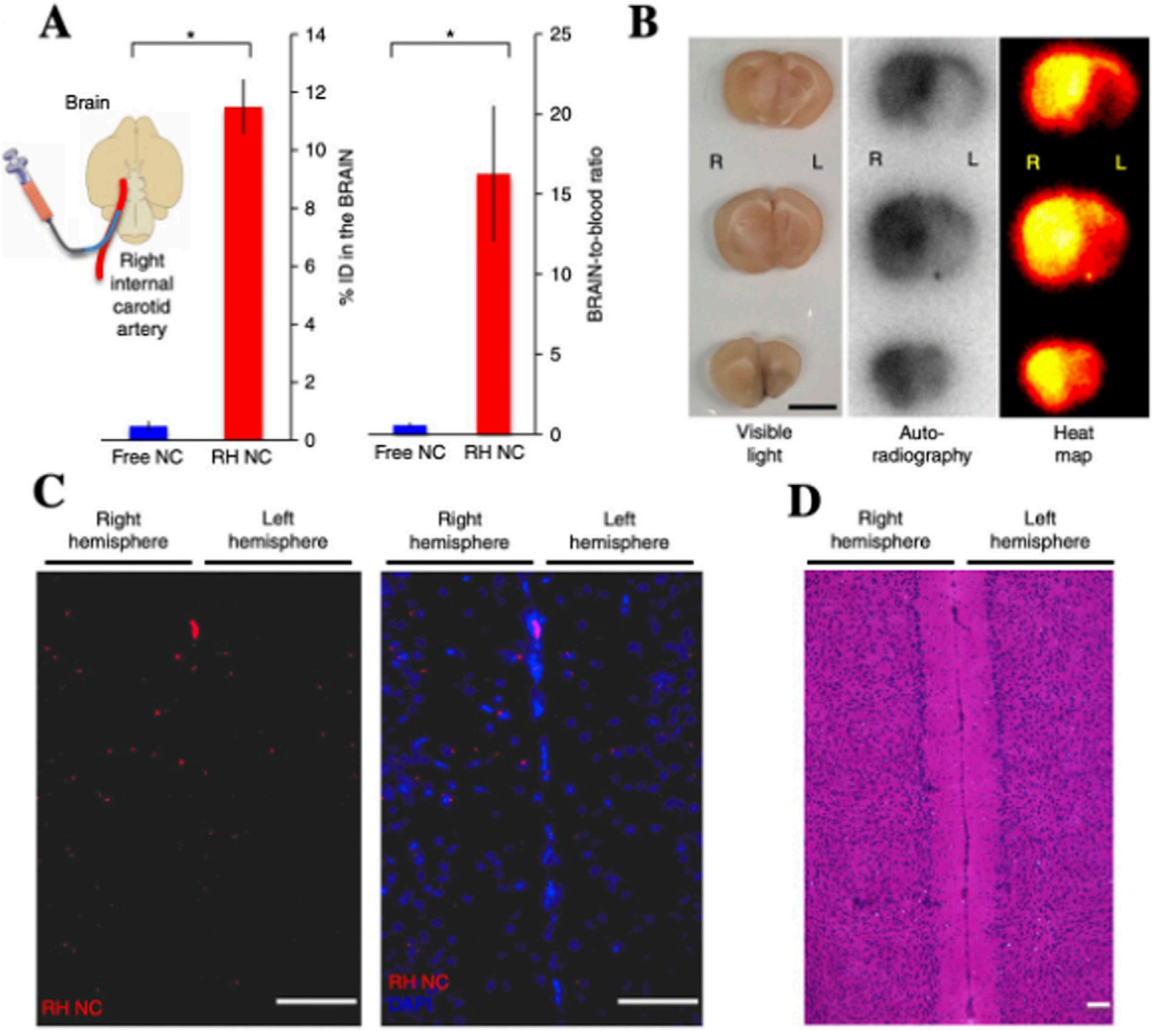
IA injection of RH-NCs enables enhanced drug delivery to downstream brain tissue. **(A)** Schematic of IA administration via right internal carotid artery to direct RH-NCs to the brain. **(B)** Autoradiographic imaging of brain sections post-injection confirms targeted NC accumulation. **(C)** Rhodamine-labeled RH-NCs show preferential distribution in the right (injected) hemisphere, indicated by increased red fluorescence. **(D)** Hematoxylin and H&E staining of brain slices from **(C)** shows no observable morphological differences between hemispheres, confirming lack of RH-NC-induced neurotoxicity (Brenner et al., 2018).

According to the principle of RH technology, mechanical shear forces encountered by RBCs during capillary transit induce the detachment of their surface-bound NPs. As a result, intravenously administered RBCs-NPs predominantly accumulate in the first capillary bed encountered-typically—the lungs. In 2018, Brenner et al. (2018). Explored whether RH technology could facilitate drug delivery to organs beyond the lungs by modifying the injection route. By administering RBCs-NPs via the right internal jugular vein, they observed that NPs achieved a brain accumulation of 11.5% of the injected dose, with liver-to-brain and blood-to-brain ratios 143-fold and 27-fold higher, respectively, compared to free NPs (Figures 3A–D).

This finding is particularly noteworthy, given that the blood-brain barrier (BBB) (Abbott et al., 2010; López-Aguirre et al., 2024) presents a major challenge for brain-targeted drug delivery. The ability to achieve such high brain accumulation suggests a potential breakthrough for central nervous system therapeutics. However, current research on employing RBCs for brain-targeted delivery remains in its infancy. The scarcity of mechanistic studies and limited exploration of delivery efficiency and safety may impede the advancement of RBCs-based strategies for central nervous system applications. Therefore, future investigations should focus on elucidating the underlying transport mechanisms, optimizing injection routes, and enhancing BBB penetration to fully harness the potential of RBCs-mediated brain drug delivery.

#### 1.2.3 Liver

The treatment of liver diseases, such as hepatitis, liver fibrosis and hepatocellular carcinoma (HCC), continues to face substantial challenges, including inefficient drug delivery, off-target effects, and systemic toxicity. Among biomimetic drug delivery systems, RBCs-EVs (Ma et al., 2023) have garnered increasing attention owing to their abundant source material, long circulation time, low immunogenicity, and natural biocompatibility. Notably, since RBCs lack nuclear and mitochondrial DNA, RBCs-EVs exhibit exceptional biosafety as drug carriers (Liu et al., 2022). Moreover, RBC-EVs demonstrate inherent liver tropism, which enables preferential hepatic accumulation while reducing off-target drug deposition, thereby minimizing systemic toxicity (Xiu et al., 2022). These properties position RBCs-EVs as one of the most promising vectors for liver-targeted drug therapy.

**FIGURE 4:**
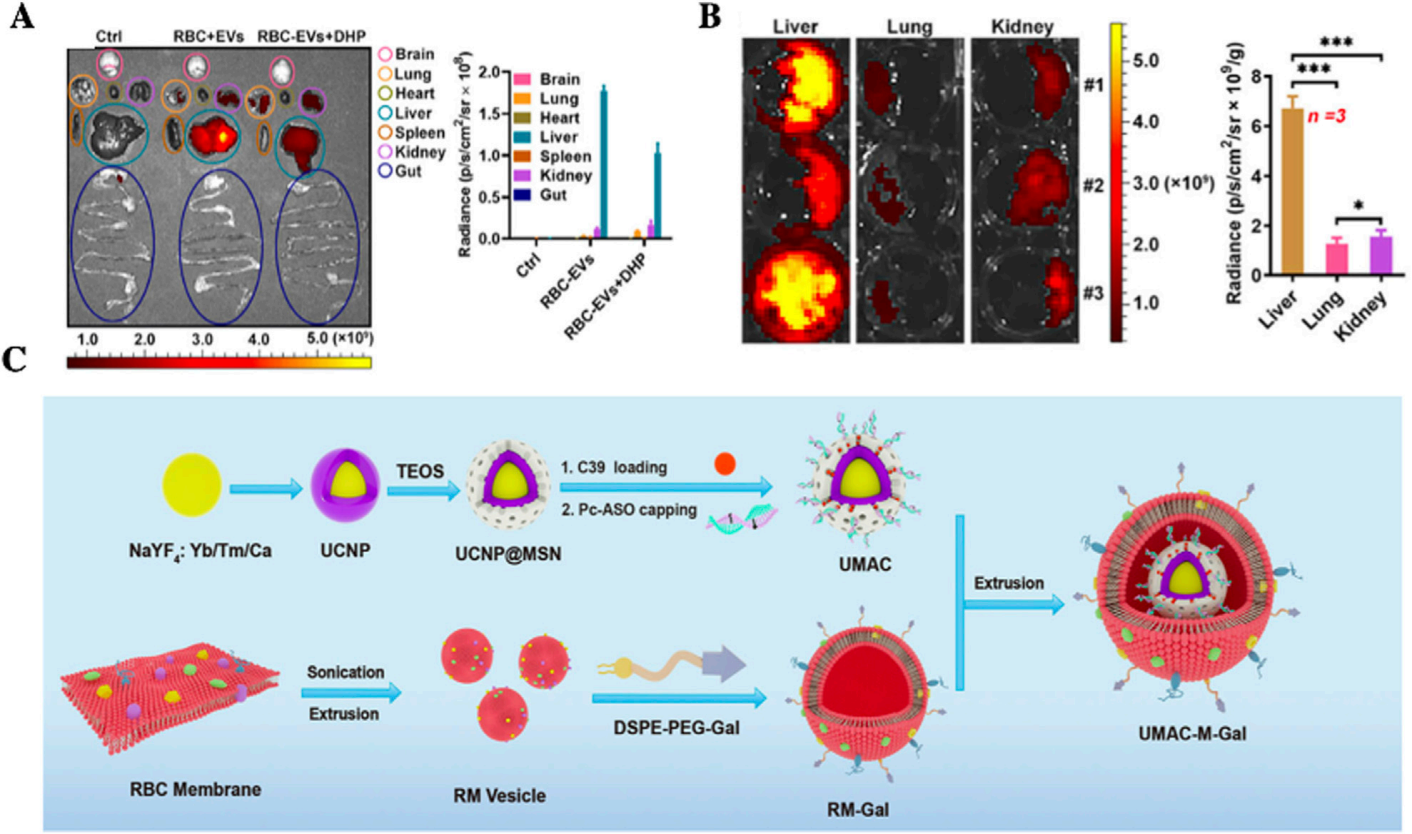
Application of RBCs-based systems for liver-targeted drug delivery. **(A)** RBC-EVs exhibit preferential liver accumulation. **(B)** Representative images and quantitative analysis of liver, lung and kidney tissues cultured with RBC-EVs for 24 h *in vitro*, highlighting enhanced hepatic uptake (Zhang et al., 2020); **(C)** Schematic illustration of an 808 nm near-infrared (NIR) light-responsive drug delivery system RBCM-UMAC, in which RBC membranes are coated onto UCNP- based nanocarriers to enable Gal-mediated liver targeting and light-controlled drug release for sustained suppression of HBV replication (Chen et al., 2023).

In a 2020 study, Zhang et al. (2020). Reported that RBCs-EVs loaded with DOX significantly enhanced therapeutic efficacy in an HCC mouse model compared to free DOX administration. Importantly, RBCs-EVs-mediated delivery result in markedly increased hepatic drug accumulation and reduced drug distribution in the lungs and spleen (Figures 4A,B). The study suggested that the liver accumulation of RBCs-EVs was predominantly mediated by hepatic macrophages, influenced by several factors: a) Liver macrophages express lower levels of signal regulatory protein α (SIRPα) compared to those in the lungs and spleen, potentially leading to diminished recognition of the CD47-mediated “do not eat me” signal; b) The liver has a high abundance of complement component C1q, which enhances macrophage phagocytosis of RBCs-EVs; and c) The liver contains a relatively large population of resident macrophages (Logtenberg et al., 2020). These mechanisms collectively facilitate efficient hepatic uptake of RBCs-EVs than promote liver-specific drug delivery. Given their high yield, favorable biosafety profile, and inherent liver-targeting properties, RBCs-EVs represent a highly promising platform for liver disease therapeutics.

Beyong RBCs-EVs, RBCM are also extensively utilized to modify drug-loaded NPs, enhancing delivery efficacy and biocompatibility (Xiong et al., 2021). Many studies (Gu et al., 2024; Hayashi et al., 2018b; Xu et al., 2023; Yang et al., 2024) have applied RBCMs as auxiliary materials to prolong systemic circulation, evade immune clearance, and minimize off-target accumulation. In 2022, Chen’s team (Chen et al., 2023) developed a light-responsive delivery system (UMAC) for hepatitis B virus (HBV) therapy. This system integrated Nd^3+^-sensitized core-shell upconversion nanoparticles (UCNPs), mesoporous silica nanoparticles (MSNs), antisense oligonucleotides (ASOs), and a capsid-binding inhibitor (C39), and was further encapsulated within RBCMs modified with β-D-galactopyranoside (Gal), thereby conferring Gal-mediated liver-targeting functionality (Figure 4C). The RBCM coating not only extended systemic circulation but also enabled precise hepatic delivery. Both *in vitro* and *in vivo* experiments demonstrated that this multifunctional platform achieved a functional cure of HBV. These findings highlight the tremendous potential of RBCs-based systems in enabling functional HBV therapy, enhancing liver-targeted drug efficacy, and providing a promising novel approach for drug delivery.

#### 1.2.4 Spleen

Following systemic administration, most NPs are phagocytosed by the RES, leading to their accumulation primarily in the liver and spleen. However, over 80% of these NPs preferentially localize in the liver, rendering the development of spleen-targeted drug delivery systems more challenging than initially anticipated (Hayashi et al., 2018a). Recent studies (Gentile et al., 2008; Luk et al., 2016; Perry et al., 2011) have highlighted that particle shape, rather than size, plays a pivotal role in macrophage-mediated phagocytosis. Notably, high-aspect-ratio particles exhibit a stronger propensity for spleen accumulation than their low-aspect-ratio counterparts. Furthermore, based on the physiological behavior of RBCs, senescent or damaged RBCs are naturally cleared by splenic macrophages and dendritic cell-an immunological process that also serves as a trigger of immune activation.

**FIGURE 5:**
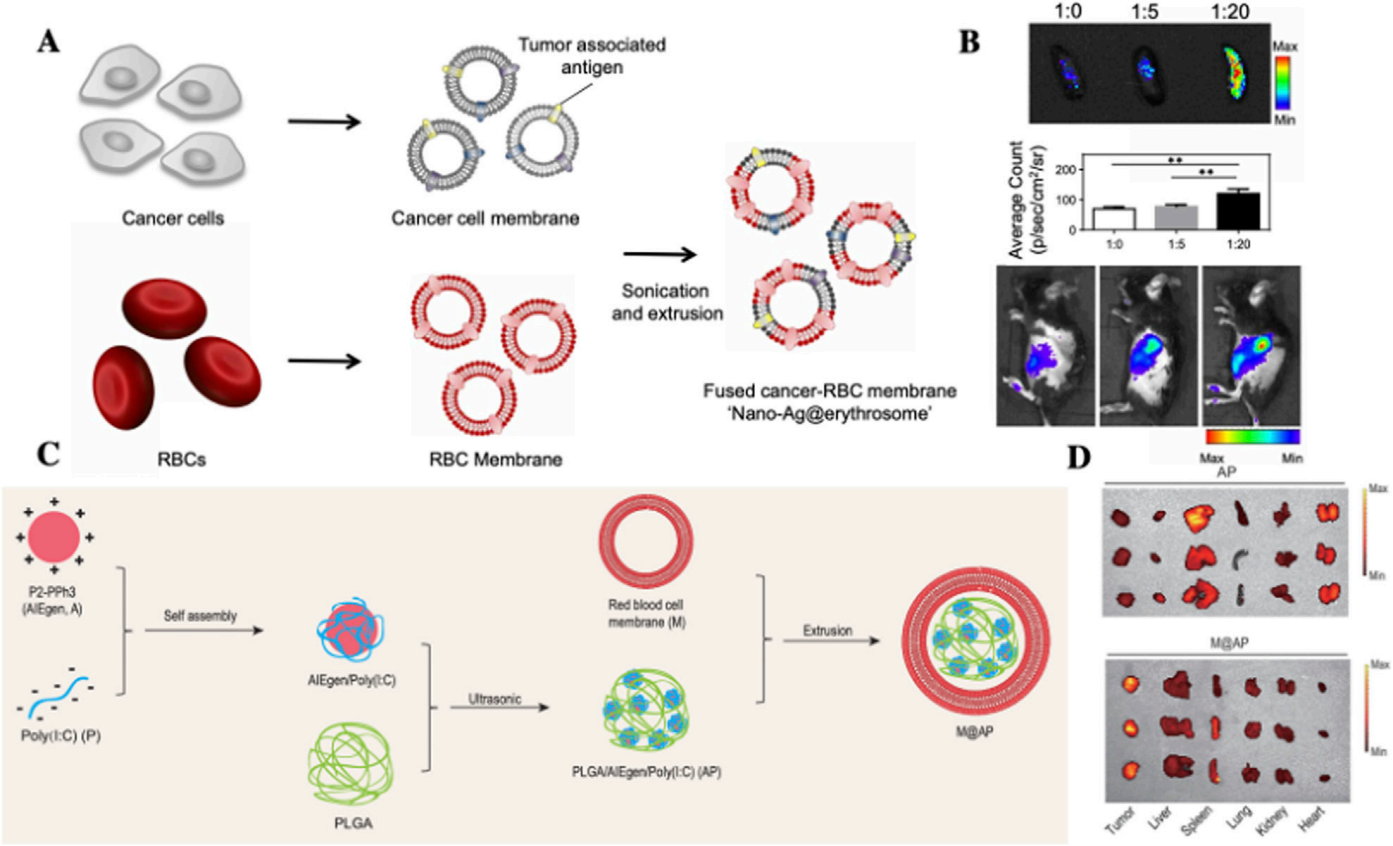
Application of RBCs-based systemsfor spleen targeted drug delivery. **(A,B)** Schematic illustration of the preparation of nano-Ag@erythrosomes by fusing TAassociated cell membranes with nano-erythrosomes. **(B)** *In vivo* fluorescence imaging of C57BL/6 mice at 1 h post-intravenous injection of nano-Ag@erythrosomes at various ratios, along with ex viv spleen imaging demonstrating ratio-dependent splenic accumulation (Han et al., 2019); **(C)** Schematic of the preparation of M@AP NPs via self-assembly of AIE photosensitizer P2PPh3 and Poly (I:C) within a PLGA matrix, followed by RBC membrane coating; **(D)** Biodistribution profiles of AP and M@AP NPs in tumor, heart, liver, spleen, lungs and kidneys, highlighting enhanced splenic and tumor accumulation mediated by RBC membrane modification (Dai et al., 2021).

Leveraging this biological mechanism, Han et al. (2019). Developed nano-Ag@erythrosomes by fusing tumor cell membrane-associated antigens with nano-erythrocytes structures to encapsulate tumor antigens (TAs) (Figures 5A,B). These nano-constructs successfully elicited antigenic immune responses *in vivo*. When administered in combination with anti-programmed death-ligand 1 (PD-L1) therapy, they significantly inhibited tumor growth in B16 F10 and 4T1 tumor models. Importantly, adjusting the RBCM-to-tumor cell membrane (R: T) ratio can modulate nano-Ag@erythrosome accumulation in kidneys or liver. Results show that varying R:T ratios minimally affect hybrid carrier size and functionality. The R:T ratio positively correlates with tumor antigen signal intensity in spleen, but not in liver or other organs. Conversely, decreasing R:T ratio promotes TAs signal accumulation in liver. The research confirms RBCM- NPs exhibit spleen-specific accumulation through homing effects, facilitating targeted TAs delivery to augment cancer immunotherapy.

This study demonstrated that RBCM-derived nano-erythrocytes effectively facilitate TAs delivery to the spleen, thereby enhancing the efficacy of cancer immunotherapy. Accordingly, RBCM-modified NPs exhibit a natural spleen-homing effect, contributing to their preferential accumulation in splenic tissue.

Building upon the unique targeting properties of RBCMs, Dai et al. (2021) developed a multifunctional nanoparticle system (M@AP-NPs) in a 2021 study (Figures 5C,D). This system was constructed these NPs through the self-assembly of a positively charged AIE photosensitizer (P2PPh3) and negatively charged Poly (I:C) within a PLGA matrix, followed by encapsulation with RBCM. *In vivo* experiments demonstrated substantial tumor accumulation of M@AP-NPs. Upon photodynamic therapy (PDT), the system not only induced direct tumor cell apoptosis but also facilitated the release of tumor antigens, thereby triggering robust antitumor immune responses. While the enhanced permeability and retention (EPR) effect primarily governed tumor targeting, the RBCM coating contributed to additional splenic accumulation through its innate homing capabilities. This dual-targeting strategy led to effective immune cell activation within the spleen, significantly augmenting systemic antitumor immunity.

Collectively, these findings underscore two key advantages of RBCM-based modification: (a) intrinsic spleen-targeting potential, and (b) immunomodulatory synergy via splenic immune activation. These attributes position RBCM-coated NPs as a promising platform for the treatment of malignancies and spleen-associated disorders.

#### 1.2.5 Whole body

**FIGURE 6:**
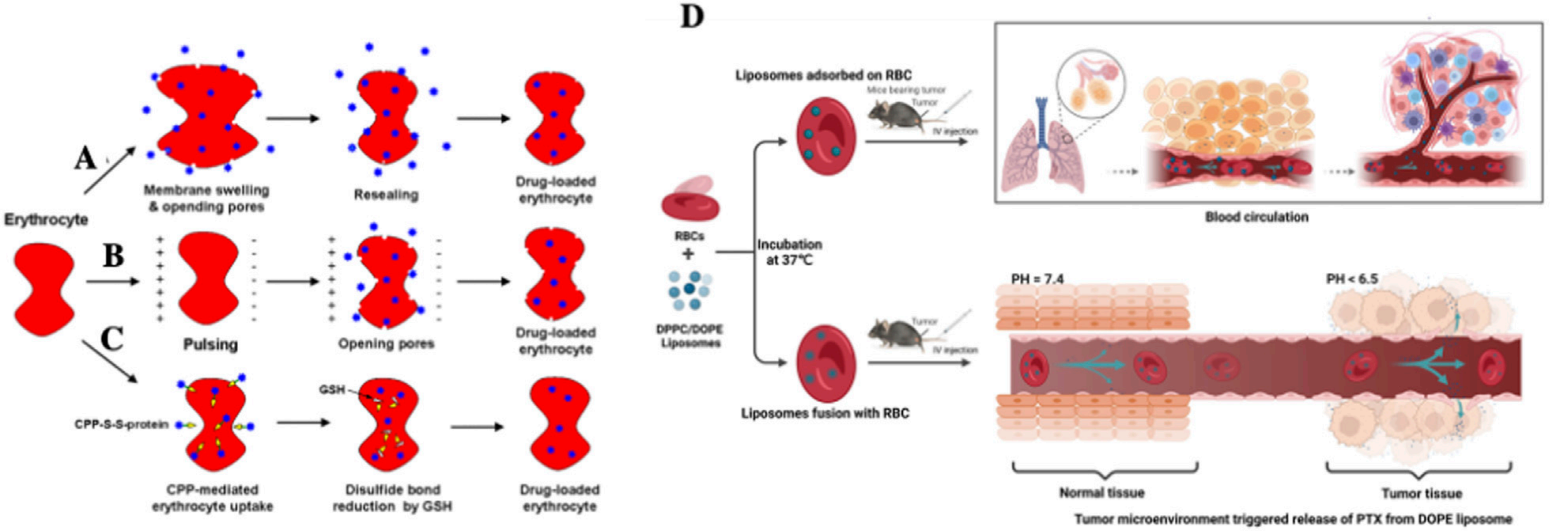
Application of RBCs-based systems for systemic drug delivery. Schematic illustration of commonly used intracellular drug loading techniques: **(A)** osmotic dialysis, **(B)** electroporation, and **(C)** CPP-mediation method (He et al., 2014). **(D)** Schematic design of a hybrid drug delivery system conbining RBCs and liposomes for enhanced anti-tumor therapy (Zhu et al., 2023).

The principal advantage of RBCs as drug carriers lies in their innate ability to evade immune clearance, reduce hepatorenal toxicity, and minimize systemic side effects, thereby enhancing the overall therapeutic efficacy of numerous RBCs-mimetic drug delivery systems (Crinelli et al., 2000; Holovati et al., 2008b; Sun et al., 2017). At present, two primary strategies are employed for systemic drug delivery using RBCs-based platforms: a) Intracellular drug loading (Brenner et al., 2021; Peng et al., 2023), which is achieved via osmotic pressure alteration (Figure 6A), induced endocytosis, or electroporation (Figure 6B). For instance, Harisa et al. (2014). Utilized this approach to encapsulate paclitaxel within RBCs, achieving an encapsulation efficiency of approximately 46.36%. Based on the same principle. EryDel’s Magnani et al. (Bourgeaux et al., 2016) developed a specialized device termed the “Red Cell Loader” to load dexamethasone-21-phosphate into RBCs for clinical applications; b) Surface conjugation (Glodek et al., 2010), wherein therapeutic agents are chemically coupled to the RBCs membrane. In a clinical study (Cowley et al., 1999) DOX-conjugated RBCs demonstrated favorable therapeutic outcomes in patients with leukemia. Similarly, JR DeLoach et al. achieved targeted lymphocyte delivery by anchoring anti-Ty-12 antibodies to RBCs surfaces.

However, both approaches present significant limitations. These manipulations often compromise RBCs membrane integrity, leading to irreversible morphological and functional alterations (Bourgeaux et al., 2016). As a result, the modified RBCs are rapidly recognized and cleared by the RES, drastically reducing drug bioavailability. Moreover, extensive surface modification can impair biocompatibility and deformability, suppress protective proteins such as CD47, or damage membrane-bound enzymes-potentially leading to the generation of reactive oxygen species (Li et al., 2020; Muzykantov, 2010; Tzounakas et al., 2017). These challenges have substantially hindered clinical translation, leaving RBCs-based delivery systems in a developmental bottleneck despite decades of research.

To overcome these limitations, researcher have explored alternative strategies to preserve RBCs integrity while maximizing their drug-carrying potential. As early as 2008, Holovati et al. (2008a) introduced a strategy to generate RBCs-lipo complexes by incubating RBCs with various concentrations of liposomes (0.25–4.0 mM) at varying temperatures (4 °C–37 °C) for durations ranging from 15 min to 6 h. The results showed that liposome binding significantly increased the PS content on the RBCs surface-approximately 100-foldcompared to free liposomes. Subsequent incubation of RBC-lipo in trehalose solution enhanced intracellular trehalose concentration by 34% ± 4%. Interestingly, liposome concentration and surface charge had minimal impact on membrane integrity, whereas incubation time and temperature were critical variables. This study demonstrated that tuning the physical properties of liposomes and incubation conditions could enable efficient RBC-liposome fusion with minimal adverse effects, offering a promising avenue for biomimetic drug delivery while maintaining native RBCs physiology.

In 2014, He et al. (2014) employed CPPs to mediate L-asparaginase loading onto RBCs surface loading of L-asparaginase loading onto RBCs. This strategy nearly doubled the enzyme’s plasma half-life in a lymphoma mouse models and significantly improved survival rates (44%) (Figure 6C). Most recently, in 2023, Zhu et al. (2023) Systematically evaluated different RBC-lipo conjugation strategies in tumor-bearing mice (Figure 6D). When liposomes adhered loosely to RBCs surfaces, analogous (similar to the RH technique principle), significant pulmonary accumulation was observed. In contrast, lipsome-RBC membrane fusion, which formed a more stable interface, extended systemic circulation time but did not enhance lung targeting. These findings suggest that therapeutic efficacy is determined by a synergistic balance between circulation longevity and organ/tissue-specific accumulation. Accordingly, the choice of conjugation method should be tailored to the therapeutic objective-whether it be systemic exposure or targeted delivery.

## 2 Conclusion

Studies show (Rossi et al., 2016; Zhang et al., 2018) that using patient-derived RBCs as drug carriers outperforms synthetic delivery systems. RBCs carriers significantly extend drug half-life *in vivo* while maintaining excellent biocompatibility and biodegradability. Their natural distribution is mainly limited to the vascular system and reticuloendothelial organs (e.g., liver and spleen). This makes RBCs ideal for delivering drugs targeting blood or these organs. As natural human components, RBCs are fully biocompatible and biodegradable. They offer clear safety advantages over nanotechnology-based systems, which often face toxicity issues. This review summarizes studies and applications of RBC-based drug delivery for organ targeting (Table 3).

**TABLE 3:** RBC-mediated organ-selective drug delivery: Target tissues, strategies, and therapeutic applications.

Organ	Target strategy	Principle	Targeted disease	Ref.
Lung	RH Technology (IV injection)	Intravenously administered NPs on RBCs surfaces are released into pulmonary capillaries	Metastatic cancer COVID-19	Anselmo et al. (2013), Liu M et al. (2024), Zelepukin et al. (2019)
				Zheng et al. (2022)
Brain	RH Technology (Right jugular vein injection)	Right-sided internal jugular vein delivery enhances brain-targeted accumulation of RBCs-NPs	Researching experiment	(Brenner et al., 2018)
Liver	RBCs-EVs	RBC-EVs accumulated in the liver	Hepatoma	Zhang et al. (2020)
	RBCM	RBCM as a targeting adjuvant material	HBV	Chen et al. (2023)
Spleen	RBCM	Homing effect of RBCM	Enhancing cancer immunotherapy	Dai et al. (2021), Han et al. (2019), Wu et al. (2025)
Whole body	Intracellular drug loading	Osmotic gradient encapsulation and electroporation	Leukemia Lymphomaetc.	Zhang et al. (2018)
	Surface conjugation	Chemical conjugation	Lymphoma	Li et al. (2024)
		Membrane fusion of RBCs and NPs	Researching experiment	Zhu et al. (2023)
	CPP	cell-penetrating peptides (CPP) capable of macromolecular transport	Lymphoma	He et al. (2014)

IV:intravenous.

Type O Rh-negative RBCs serve as natural universal carriers, compatible with nearly all blood types without rejection concerns. A single RBCs can carry millions of small molecules, enabling sufficient drug loading for *in vivo* therapeutic applications (Yu et al., 2020). For autologous therapy, processing patient-derived RBCs *ex vivo*, drug loading, and reinfusion enable enhanced therapeutic effects with lower drug doses (Biagiotti et al., 2011). This approach not only improves treatment efficacy but also advances autologous blood-based therapies.

Despite decades of research, RBC-based drug delivery systems have not yet achieved widespread clinical adoption due to multiple challenges (Blumberg et al., 2019; Dutra et al., 2023): From the perspective of transfusion-transmitted infections, historical outbreaks—such as HIV in the 1960s and increased transfusion risks from malaria, bacterial contamination, and hepatitis in the 1970s—were primarily due to improper donor selection and inadequate blood processing. Modern transfusion medicine has greatly reduced these risks through rigorous donor/recipient screening (e.g., for HIV, syphilis, hepatitis) and the development of pathogen inactivation techniques, such as those applied to clotting factor concentrates. However, the problem is not fully resolved. New pathogens continue to emerge—as seen with COVID-19 — which disrupted healthcare systems and reduced eligible donor pools (Noordin et al., 2021; Raturi and Kusum, 2020). Therefore, the risk of transfusion-transmitted infections remains a persistent and critical challenge in the development and clinical translation of RBC-based therapeutics.

Secondly, allogeneic RBCs delivery involves complex issues including donor sourcing, storage requirements, preparation standardization, and ethical considerations. Patient-derived RBCs often exhibit structural, molecular, or rheological differences from healthy RBCs that can alter drug pharmacokinetics, resulting in significant batch-to-batch variability. This biological inconsistency creates greater regulatory hurdles than well-defined synthetic carriers. Studies by Sarah Costantino et al. demonstrated that RBCs-EVs infusion induces endothelial dysfunction in type 2 diabetes patients, revealing unresolved safety limitations of RBCs-EVs carriers and underscoring the translational challenges of RBCs delivery platforms. Notably, the shelf life of isolated RBCs is 35 days, and drug-loaded RBCs generally require fresh preparation, whereas most synthetic materials have longer durability. Thus, while RBCs-based drug delivery systems demonstrate clear advantages in prolonged circulation and biosafety, challenges remain in large-scale production and broad drug applicability.

The organ-targeting specificity of RBCs-based drug delivery systems is coordinately regulated by multiple factors. The injection site serves as a critical determinant of initial biodistribution, with right jugular vein injection significantly enhancing brain targeting efficiency while tail vein injection preferentially enriches liver/spleen accumulation (Brenner et al., 2021; Brenner et al., 2018). Surface modifications enable precise targeting through specific molecular interactions, being particularly suitable for rapid drug release (e.g., mononuclear phagocyte system targeting in spleen/lung) albeit with relatively lower drug loading capacity; Intracellular encapsulation markedly prolongs systemic circulation time for sustained drug release, yet this approach more readily compromises native RBCs characteristics and demands more sophisticated loading techniques; Membrane fusion technology allows modulation of targeting specificity and pharmacokinetics through NPs composition adjustments (Favretto et al., 2013; Hadi Barhaghtalab et al., 2024; Wibroe et al., 2017). Optimal targeting strategies require integrated consideration of administration routes (e.g., right jugular vein), engineered modifications (e.g., organ-specific ligands), loading methods (surface adsorption vs. intracellular encapsulation). These findings elucidate the molecular basis for organ-specific delivery using RBCs-based systems and provide theoretical foundations for designing precision drug administration regimens in clinical translation.

In parallel, blood cell-derived biomimetic carriers have garnered increasing interest as promising vehicles for targeted therapy. Novel strategies have emerged that integrate the benefits of multiple blood components to enhance delivery efficacy. For example, hybrid systems incorporating RBCM and platelet membranes have been developed to simultaneously reduce immunogenicity and facilitate tumor-specific targeting (Dehaini et al., 2017); Similarly, RBCs-tumor cell membrane hybrids exploit tumor homing mechanisms for precise lesion localization (Xiong et al., 2021).

In conclusion, while notable challenges persist, the multi-organ targeting capability of RBCs-based drug delivery systems remains a distinctive and advantageous feature. With continued advancements in bioengineering and nanotechnology, further exploration and refinement of RBCs-mediated platforms are warranted to address current limitations and advance their clinical applications.

## References

[B1] Abbott N. J. Patabendige A. A. Dolman D. E. Yusof S. R. Begley D. J. (2010). Structure and function of the blood-brain barrier. Neurobiol. Dis. 37 (1), 13–25. 10.1016/j.nbd.2009.07.030 19664713

[B2] Adepu S. Ramakrishna S. (2021). Controlled drug delivery systems: current status and future directions. Molecules 26 (19), 5905. 10.3390/molecules26195905 34641447 PMC8512302

[B3] Agrawal V. Woo J. H. Borthakur G. Kantarjian H. Frankel A. E. (2013). Red blood cell-encapsulated L-asparaginase: potential therapy of patients with asparagine synthetase deficient acute myeloid leukemia. Protein Pept. Lett. 20 (4), 392–402. 23016580

[B4] Akbas E. M. Akbas N. (2021). COVID-19, adrenal gland, glucocorticoids, and adrenal insufficiency. Biomed. Pap. Med. Fac. Univ. Palacky. Olomouc Czech Repub. 165 (1), 1–7. 10.5507/bp.2021.011 33542545

[B5] Alanazi F. K. Harisa Gel D. Maqboul A. Abdel-Hamid M. Neau S. H. Alsarra I. A. (2011). Biochemically altered human erythrocytes as a carrier for targeted delivery of primaquine: an *in vitro* study. Arch. Pharm. Res. 34 (4), 563–571. 10.1007/s12272-011-0406-7 21544721

[B6] Alexaki V. I. Henneicke H. (2021). The role of glucocorticoids in the management of COVID-19. Horm. Metab. Res. 53 (1), 9–15. 10.1055/a-1300-2550 33207372 PMC7781662

[B7] Amreddy N. Babu A. Muralidharan R. Panneerselvam J. Srivastava A. Ahmed R. (2018). Recent advances in nanoparticle-based cancer drug and gene delivery. Adv. Cancer Res. 137, 115–170. 10.1016/bs.acr.2017.11.003 29405974 PMC6550462

[B8] Anselmo A. C. Gupta V. Zern B. J. Pan D. Zakrewsky M. Muzykantov V. (2013). Delivering nanoparticles to lungs while avoiding liver and spleen through adsorption on red blood cells. ACS Nano 7 (12), 11129–11137. 10.1021/nn404853z 24182189 PMC4128963

[B9] Beach M. A. Nayanathara U. Gao Y. Zhang C. Xiong Y. Wang Y. (2024). Polymeric nanoparticles for drug delivery. Chem. Rev. 124 (9), 5505–5616. 10.1021/acs.chemrev.3c00705 38626459 PMC11086401

[B10] Belov A. Yang H. Forshee R. A. Whitaker B. I. Eder A. F. Chancey C. (2023). Modeling the risk of HIV transfusion transmission. J. Acquir Immune Defic. Syndr. 92 (2), 173–179. 10.1097/qai.0000000000003115 36219691

[B11] Biagiotti S. Paoletti M. F. Fraternale A. Rossi L. Magnani M. (2011). Drug delivery by red blood cells. IUBMB Life 63 (8), 621–631. 10.1002/iub.478 21766411

[B12] Blumberg N. Cholette J. M. Cahill C. Pietropaoli A. P. Winters S. Phipps R. (2019). Transfusion medicine: a research agenda for the coming years. Transfus. Apher. Sci. 58 (5), 698–700. 10.1016/j.transci.2019.08.015 31402101

[B13] Bourgeaux V. Lanao J. M. Bax B. E. Godfrin Y. (2016). Drug-loaded erythrocytes: on the road toward marketing approval. Drug Des. Devel Ther. 10, 665–676. 10.2147/DDDT.S96470 26929599 PMC4755692

[B14] Brenner J. S. Pan D. C. Myerson J. W. Marcos-Contreras O. A. Villa C. H. Patel P. (2018). Red blood cell-hitchhiking boosts delivery of nanocarriers to chosen organs by orders of magnitude. Nat. Commun. 9 (1), 2684. 10.1038/s41467-018-05079-7 29992966 PMC6041332

[B15] Brenner J. S. Mitragotri S. Muzykantov V. R. (2021). Red blood cell hitchhiking: a novel approach for vascular delivery of nanocarriers. Annu. Rev. Biomed. Eng. 23, 225–248. 10.1146/annurev-bioeng-121219-024239 33788581 PMC8277719

[B16] Carrasco-Sánchez V. Vergara-Jaque A. Zuñiga M. Comer J. John A. Nachtigall F. M. (2014). *In situ* and *in silico* evaluation of amine- and folate-terminated dendrimers as nanocarriers of anesthetics. Eur. J. Med. Chem. 73, 250–257. 10.1016/j.ejmech.2013.11.040 24412500

[B17] Chen Z. A. Wu S. H. Chen P. Chen Y. P. Mou C. Y. (2019). Critical Features for mesoporous silica nanoparticles encapsulated into erythrocytes. ACS Appl. Mater Interfaces 11 (5), 4790–4798. 10.1021/acsami.8b18434 30624037

[B18] Chen S. Song Z. Feng R. (2020). Recent development of Copolymeric nano-drug delivery system for paclitaxel. Anticancer Agents Med. Chem. 20 (18), 2169–2189. 10.2174/1871520620666200719001038 32682385

[B19] Chen L. Jiang X. Liu Q. Tang Z. Wang D. Xiang Z. (2023). A dual-targeting near-infrared biomimetic drug delivery system for HBV treatment. J. Med. Virol. 95 (1), e28312. 10.1002/jmv.28312 36404678

[B20] Cheng R. Wang S. (2024). Cell-mediated nanoparticle delivery systems: towards precision nanomedicine. Drug Deliv. Transl. Res. 14 (11), 3032–3054. 10.1007/s13346-024-01591-0 38615157 PMC11445310

[B21] Cheng Y. H. He C. Riviere J. E. Monteiro-Riviere N. A. Lin Z. (2020). Meta-analysis of nanoparticle delivery to tumors using a physiologically based pharmacokinetic modeling and Simulation approach. ACS Nano 14 (3), 3075–3095. 10.1021/acsnano.9b08142 32078303 PMC7098057

[B22] Choi A. Javius-Jones K. Hong S. Park H. (2023). Cell-based drug delivery systems with innate homing capability as a novel nanocarrier platform. Int. J. Nanomedicine 18, 509–525. 10.2147/IJN.S394389 36742991 PMC9893846

[B23] Cowley H. Wojda U. Cipolone K. M. Procter J. L. Stroncek D. F. Miller J. L. (1999). Biotinylation modifies red cell antigens. Transfusion 39 (2), 163–168. 10.1046/j.1537-2995.1999.39299154730.x 10037126

[B24] Crinelli R. Antonelli A. Bianchi M. Gentilini L. Scaramucci S. Magnani M. (2000). Selective inhibition of NF-kB activation and TNF-alpha production in macrophages by red blood cell-mediated delivery of dexamethasone. Blood Cells Mol. Dis. 26 (3), 211–222. 10.1006/bcmd.2000.0298 10950941

[B25] da Silveira Cavalcante L. Feng Q. Chin-Yee I. Acker J. P. Holovati J. L. (2017). Effect of liposome-treated red blood cells in an anemic rat model. J. Liposome Res. 27 (1), 56–63. 10.3109/08982104.2016.1149867 27055898

[B26] da Silveira Cavalcante L. Branch D. R. Duong T. T. Yeung R. S. M. Acker J. P. Holovati J. L. (2018). The immune-stimulation capacity of liposome-treated red blood cells. J. Liposome Res. 28 (3), 173–181. 10.1080/08982104.2017.1295991 28276279

[B27] Dabagh S. Haris S. A. Ertas Y. N. (2023). Engineered polyethylene glycol-coated Zinc Ferrite nanoparticles as a novel Magnetic Resonance imaging contrast agent. ACS Biomater. Sci. Eng. 9 (7), 4138–4148. 10.1021/acsbiomaterials.3c00255 37311018 PMC10336746

[B28] Dai J. Wu M. Wang Q. Ding S. Dong X. Xue L. (2021). Red blood cell membrane-camouflaged nanoparticles loaded with AIEgen and Poly(I: C) for enhanced tumoral photodynamic-immunotherapy. Natl. Sci. Rev. 8 (6), nwab039. 10.1093/nsr/nwab039 34691671 PMC8288176

[B29] de Lemos Neto M. Alexandre R. C. V. Morra R. O. G. da Paz J. A. S. Barroso S. P. C. Resende A. C. (2021). Use of glucocorticoids and azithromycin in the therapy of COVID-19. Pharmacol. Rep. 73 (6), 1513–1519. 10.1007/s43440-021-00286-4 34085181 PMC8175191

[B30] Dehaini D. Wei X. Fang R. H. Masson S. Angsantikul P. Luk B. T. (2017). Erythrocyte-platelet hybrid membrane coating for enhanced nanoparticle functionalization. Adv. Mater 29 (16), 1606209. 10.1002/adma.201606209 28199033 PMC5469720

[B31] Della Pelle G. Kostevsek N. (2021). Nucleic acid delivery with red-blood-cell-based carriers. Int. J. Mol. Sci. 22 (10), 5264. 10.3390/ijms22105264 34067699 PMC8156122

[B32] Di Y. Wang W. Wang Y. Wang J. (2023). Recent engineering advances of EVs for compounds, nucleic acids, and TCM delivery. Eur. J. Pharm. Sci. 190, 106584. 10.1016/j.ejps.2023.106584 37717667

[B33] Dutra V. F. Bonet-Bub C. Sakashita A. M. Kutner J. M. (2023). Infectious diseases and the impact on transfusion medicine: a historical review and lessons for the future. Transfus. Clin. Biol. 30 (4), 376–381. 10.1016/j.tracli.2023.06.004 37328129

[B34] Ebrahimi S. Bagchi P. (2022). A computational study of red blood cell deformability effect on hemodynamic alteration in capillary vessel networks. Sci. Rep. 12 (1), 4304. 10.1038/s41598-022-08357-z 35277592 PMC8917159

[B35] Favretto M. E. Cluitmans J. C. Bosman G. J. Brock R. (2013). Human erythrocytes as drug carriers: loading efficiency and side effects of hypotonic dialysis, chlorpromazine treatment and fusion with liposomes. J. Control Release 170 (3), 343–351. 10.1016/j.jconrel.2013.05.032 23747798

[B36] Gardos G. (1954). Accumulation of potassium ions by human blood corpuscles. Acta Physiol. Acad. Sci. Hung 6 (2-3), 191–199. 13217849

[B37] Gautam M. Jozic A. Su G. L. Herrera-Barrera M. Curtis A. Arrizabalaga S. (2023). Lipid nanoparticles with PEG-variant surface modifications mediate genome editing in the mouse retina. Nat. Commun. 14 (1), 6468. 10.1038/s41467-023-42189-3 37833442 PMC10575971

[B38] Gentile F. Chiappini C. Fine D. Bhavane R. C. Peluccio M. S. Cheng M. M. (2008). The effect of shape on the margination dynamics of non-neutrally buoyant particles in two-dimensional shear flows. J. Biomech. 41 (10), 2312–2318. 10.1016/j.jbiomech.2008.03.021 18571181

[B39] Ginn F. L. Hochstein P. Trump B. F. (1969). Membrane alterations in hemolysis: Internalization of plasmalemma induced by primaquine. Science 164 (3881), 843–845. 10.1126/science.164.3881.843 5767788

[B40] Glodek A. M. Mirchev R. Golan D. E. Khoory J. A. Burns J. M. Shevkoplyas S. S. (2010). Ligation of complement receptor 1 increases erythrocyte membrane deformability. Blood 116 (26), 6063–6071. 10.1182/blood-2010-04-273904 20861458 PMC3031392

[B41] Gu J. Yan C. Yin S. Wu H. Liu C. Xue A. (2024). Erythrocyte membrane-coated nanocarriers modified by TGN for Alzheimer's disease. J. Control Release 366, 448–459. 10.1016/j.jconrel.2023.12.030 38128884

[B42] Gutierrez-Millan C. Barez Diaz C. Alvarez Vizan L. Colino C. I. (2023). Evaluation of two Osmosis-based methods for the preparation of drug delivery systems based on red blood cells. Pharmaceutics 15 (9), 2281. 10.3390/pharmaceutics15092281 37765250 PMC10536362

[B43] Hadi Barhaghtalab R. Tanimowo Aiyelabegan H. Maleki H. Mirzavi F. Gholizadeh Navashenaq J. Abdi F. (2024). Recent advances with erythrocytes as therapeutics carriers. Int. J. Pharm. 665, 124658. 10.1016/j.ijpharm.2024.124658 39236775

[B44] Han X. Shen S. Fan Q. Chen G. Archibong E. Dotti G. (2019). Red blood cell-derived nanoerythrosome for antigen delivery with enhanced cancer immunotherapy. Sci. Adv. 5 (10), eaaw6870. 10.1126/sciadv.aaw6870 31681841 PMC6810293

[B45] Harisa G. I. Ibrahim M. F. Alanazi F. Shazly G. A. (2014). Engineering erythrocytes as a novel carrier for the targeted delivery of the anticancer drug paclitaxel. Saudi Pharm. J. 22 (3), 223–230. 10.1016/j.jsps.2013.06.007 25061408 PMC4099562

[B46] Harisa Gel D. Ibrahim M. F. Alanazi F. K. (2011). Characterization of human erythrocytes as potential carrier for pravastatin: an *in vitro* study. Int. J. Med. Sci. 8 (3), 222–230. 10.7150/ijms.8.222 21448309 PMC3065791

[B47] Hayashi K. Yamada S. Hayashi H. Sakamoto W. Yogo T. (2018a). Red blood cell-like particles with the ability to avoid lung and spleen accumulation for the treatment of liver fibrosis. Biomaterials 156, 45–55. 10.1016/j.biomaterials.2017.11.031 29190497

[B48] Hayashi K. Yamada S. Sakamoto W. Usugi E. Watanabe M. Yogo T. (2018b). Red blood cell-Shaped Microparticles with a red blood cell membrane demonstrate prolonged circulation time in blood. ACS Biomater. Sci. Eng. 4 (8), 2729–2732. 10.1021/acsbiomaterials.8b00197 33434998

[B49] He H. Ye J. Wang Y. Liu Q. Chung H. S. Kwon Y. M. (2014). Cell-penetrating peptides meditated encapsulation of protein therapeutics into intact red blood cells and its application. J. Control Release 176, 123–132. 10.1016/j.jconrel.2013.12.019 24374002 PMC3939723

[B50] He J. Zhang X. Liu L. Wang Y. Liu R. Li M. (2023). Acute and Subacute toxicity evaluation of erythrocyte membrane-coated Boron Nitride nanoparticles. J. Funct. Biomater. 14 (4), 181. 10.3390/jfb14040181 37103271 PMC10144386

[B51] He Y. Wang Y. Wang L. Jiang W. Wilhelm S. (2024). Understanding nanoparticle-liver interactions in nanomedicine. Expert Opin. Drug Deliv. 21 (6), 829–843. 10.1080/17425247.2024.2375400 38946471 PMC11281865

[B52] Hima F. Kalverkamp S. Kashefi A. Mottaghy K. Zayat R. Strudthoff L. (2023). Oxygenation performance assessment of an artificial lung in different central anatomic configurations. Int. J. Artif. Organs 46 (5), 295–302. 10.1177/03913988231168163 37051677 PMC10160396

[B53] Holovati J. L. Gyongyossy-Issa M. I. C. Acker J. P. (2008a). Effect of liposome charge and composition on the delivery of trehalose into red blood cells. Cell Preserv. Technol. 6 (3), 207–218. 10.1089/cpt.2008.0008

[B54] Holovati J. L. Gyongyossy-Issa M. I. C. Acker J. P. (2008b). Investigating interactions of trehalose-containing liposomes with human red blood cells. Cell Preserv. Technol. 6 (2), 133–146. 10.1089/cpt.2008.0004

[B55] Hou K. Zhang Y. Bao M. Xin C. Wei Z. Lin G. (2022). A multifunctional Magnetic red blood cell-mimetic Micromotor for drug delivery and Image-Guided therapy. ACS Appl. Mater Interfaces 14 (3), 3825–3837. 10.1021/acsami.1c21331 35025195

[B56] Hu B. Guo H. Zhou P. Shi Z. L. (2021). Characteristics of SARS-CoV-2 and COVID-19. Nat. Rev. Microbiol. 19 (3), 141–154. 10.1038/s41579-020-00459-7 33024307 PMC7537588

[B57] Huynh T. M. H. Yalamandala B. N. Chiang M. R. Weng W. H. Chang C. W. Chiang W. H. (2023). Programmed antigen capture-harnessed dendritic cells by margination-hitchhiking lung delivery. J. Control Release 358, 718–728. 10.1016/j.jconrel.2023.05.028 37230295

[B58] Ji W. Smith P. N. Koepsel R. R. Andersen J. D. Baker S. L. Zhang L. (2020). Erythrocytes as carriers of immunoglobulin-based therapeutics. Acta Biomater. 101, 422–435. 10.1016/j.actbio.2019.10.027 31669698

[B59] Jia B. Shi Y. Yan Y. Shi H. Zheng J. Liu J. (2025). Engineering of erythrocytes as drug carriers for therapeutic applications. Adv. Biol. (Weinh) 9 (5), 2400242. 10.1002/adbi.202400242 39037400

[B60] Krivic H. Himbert S. Rheinstadter M. C. (2022). Perspective on the application of erythrocyte liposome-based drug delivery for infectious diseases. Membr. (Basel) 12 (12), 1226. 10.3390/membranes12121226 36557133 PMC9785899

[B61] Lehmann T. P. Golik M. Olejnik J. Łukaszewska M. Markowska D. Drożdżyńska M. (2023). Potential applications of using tissue-specific EVs in targeted therapy and vaccinology. Biomed. Pharmacother. 166, 115308. 10.1016/j.biopha.2023.115308 37660644

[B62] Li C. Xie Z. Chen Q. Zhang Y. Chu Y. Guo Q. (2020). Supramolecular Hunter Stationed on red blood cells for Detoxification based on specific molecular recognition. ACS Nano 14 (4), 4950–4962. 10.1021/acsnano.0c01119 32203660

[B63] Li B. Shao H. Gao L. Li H. Sheng H. Zhu L. (2022). Nano-drug co-delivery system of natural active ingredients and chemotherapy drugs for cancer treatment: a review. Drug Deliv. 29 (1), 2130–2161. 10.1080/10717544.2022.2094498 35815678 PMC9275501

[B64] Li B. Yuan D. Chen H. Wang X. Liang Y. Wong C. T. T. (2024). Site-selective antibody-lipid conjugates for surface functionalization of red blood cells and targeted drug delivery. J. Control Release 370, 302–309. 10.1016/j.jconrel.2024.04.038 38663752

[B65] Liang X. Li H. Zhang A. Tian X. Guo H. Zhang H. (2022). Red blood cell biomimetic nanoparticle with anti-inflammatory, anti-oxidative and hypolipidemia effect ameliorated atherosclerosis therapy. Nanomedicine 41, 102519. 10.1016/j.nano.2022.102519 35038590

[B66] Lin Y. C. Chen B. M. Tran T. T. M. Chang T. C. Al-Qaisi T. S. Roffler S. R. (2023). Accelerated clearance by antibodies against methoxy PEG depends on pegylation architecture. J. Control Release 354, 354–367. 10.1016/j.jconrel.2023.01.021 36641121

[B67] Liu Z. Chan R. B. Cai Z. Liu X. Wu Y. Yu Z. (2022). α-Synuclein-containing erythrocytic extracellular vesicles: essential contributors to hyperactivation of monocytes in Parkinson’s disease. J. Neuroinflammation 19 (1), 53. 10.1186/s12974-022-02413-1 35193594 PMC8862590

[B68] Liu M. Zhang R. Huang H. Liu P. Zhao X. Wu H. (2024). Erythrocyte-leveraged oncolytic virotherapy (ELeOVt): oncolytic virus assembly on erythrocyte surface to Combat pulmonary metastasis and Alleviate side effects. Adv. Sci. (Weinh) 11 (5), 2303907. 10.1002/advs.202303907 37997186 PMC10837356

[B69] Liu W. Liu L. Li H. Xie Y. Bai J. Guan J. (2024). Targeted pathophysiological treatment of ischemic stroke using nanoparticle-based drug delivery system. J. Nanobiotechnology 22 (1), 499. 10.1186/s12951-024-02772-2 39164747 PMC11337765

[B70] Logtenberg M. E. W. Scheeren F. A. Schumacher T. N. (2020). The CD47-SIRPα immune Checkpoint. Immunity 52 (5), 742–752. 10.1016/j.immuni.2020.04.011 32433947 PMC7340539

[B71] López-Aguirre M. Castillo-Ortiz M. Viña-González A. Blesa J. Pineda-Pardo J. A. (2024). The road ahead to successful BBB opening and drug-delivery with focused ultrasound. J. Control Release 372, 901–913. 10.1016/j.jconrel.2024.07.006 38971426

[B72] Lucas A. Lam D. Cabrales P. (2019). Doxorubicin-loaded red blood cells reduced cardiac toxicity and preserved anticancer activity. Drug Deliv. 26 (1), 433–442. 10.1080/10717544.2019.1591544 30929538 PMC6450495

[B73] Luk B. T. Fang R. H. Hu C. M. Copp J. A. Thamphiwatana S. Dehaini D. (2016). Safe and Immunocompatible nanocarriers Cloaked in RBC membranes for drug delivery to treat Solid tumors. Theranostics 6 (7), 1004–1011. 10.7150/thno.14471 27217833 PMC4876624

[B74] Ma S. R. Xia H. F. Gong P. Yu Z. L. (2023). Red blood cell-derived extracellular vesicles: an overview of current research progress, challenges, and Opportunities. Biomedicines 11 (10), 2798. 10.3390/biomedicines11102798 37893171 PMC10604118

[B75] Mahmood A. Munir T. Rasul A. Ghfar A. A. Mumtaz S. (2023). Polyethylene glycol and chitosan functionalized manganese oxide nanoparticles for antimicrobial and anticancer activities. J. Colloid Interface Sci. 648, 907–915. 10.1016/j.jcis.2023.06.029 37329602

[B76] Mambrini G. Mandolini M. Rossi L. Pierige F. Capogrossi G. Salvati P. (2017). *Ex vivo* encapsulation of dexamethasone sodium phosphate into human autologous erythrocytes using fully automated biomedical equipment. Int. J. Pharm. 517 (1-2), 175–184. 10.1016/j.ijpharm.2016.12.011 27939571

[B77] Meng W. He C. Hao Y. Wang L. Li L. Zhu G. (2020). Prospects and challenges of extracellular vesicle-based drug delivery system: considering cell source. Drug Deliv. 27 (1), 585–598. 10.1080/10717544.2020.1748758 32264719 PMC7178886

[B78] Mitchell D. H. James G. T. Kruse C. A. (1990). Bioactivity of electric field-pulsed human recombinant interleukin-2 and its encapsulation into erythrocyte carriers. Biotechnol. Appl. Biochem. 12 (3), 264–275. 10.1111/j.1470-8744.1990.tb00099.x 2360992

[B79] Muzykantov V. R. (2010). Drug delivery by red blood cells: vascular carriers designed by mother nature. Expert Opin. Drug Deliv. 7 (4), 403–427. 10.1517/17425241003610633 20192900 PMC2844929

[B80] Nguyen P. H. D. Jayasinghe M. K. Le A. H. Peng B. Le M. T. N. (2023). Advances in drug delivery systems based on red blood cells and their membrane-derived nanoparticles. ACS Nano 17 (6), 5187–5210. 10.1021/acsnano.2c11965 36896898

[B81] Nie Y. Fu G. Leng Y. (2023). Nuclear delivery of nanoparticle-based drug delivery systems by nuclear localization signals. Cells 12 (12), 1637. 10.3390/cells12121637 37371107 PMC10297004

[B82] Noordin S. S. Yusoff N. M. Karim F. A. Chong S. E. (2021). Blood transfusion services amidst the COVID-19 pandemic. J. Glob. Health 11, 03053. 10.7189/jogh.11.03053 33884188 PMC8053396

[B83] Peng W. Yue Y. Zhang Y. Li H. Zhang C. Wang P. (2023). Scheduled dosage regimen by irreversible electroporation of loaded erythrocytes for cancer treatment. Apl. Bioeng. 7 (4), 046102. 10.1063/5.0174353 37854061 PMC10581719

[B84] Perry J. L. Herlihy K. P. Napier M. E. Desimone J. M. (2011). PRINT: a novel platform toward shape and size specific nanoparticle theranostics. Acc. Chem. Res. 44 (10), 990–998. 10.1021/ar2000315 21809808 PMC4157651

[B85] Puidokas T. Kubilius M. Stumbras A. Juodzbalys G. (2019). Effect of leukocytes included in platelet concentrates on cell behaviour. Platelets 30 (8), 937–945. 10.1080/09537104.2019.1646900 31340699

[B86] Raturi M. Kusum A. (2020). The blood supply management amid the COVID-19 outbreak. Transfus. Clin. Biol. 27 (3), 147–151. 10.1016/j.tracli.2020.04.002 32386966 PMC7194633

[B87] Reale R. De Ninno A. Nepi T. Bisegna P. Caselli F. (2023). Extensional-flow impedance Cytometer for Contactless and Optics-free erythrocyte deformability analysis. IEEE Trans. Biomed. Eng. 70 (2), 565–572. 10.1109/tbme.2022.3197214 35939464

[B88] Ricciotti E. Laudanski K. FitzGerald G. A. (2021). Nonsteroidal anti-inflammatory drugs and glucocorticoids in COVID-19. Adv. Biol. Regul. 81, 100818. 10.1016/j.jbior.2021.100818 34303107 PMC8280659

[B89] Rossi L. Pierige F. Antonelli A. Bigini N. Gabucci C. Peiretti E. (2016). Engineering erythrocytes for the modulation of drugs' and contrasting agents' pharmacokinetics and biodistribution. Adv. Drug Deliv. Rev. 106 (Pt A), 73–87. 10.1016/j.addr.2016.05.008 27189231

[B90] Saadati R. Dadashzadeh S. Abbasian Z. Soleimanjahi H. (2013). Accelerated blood clearance of PEGylated PLGA nanoparticles following repeated injections: effects of polymer dose, PEG coating, and encapsulated anticancer drug. Pharm. Res. 30 (4), 985–995. 10.1007/s11095-012-0934-y 23184228

[B91] Sangha G. S. Weber C. M. Sapp R. M. Setua S. Thangaraju K. Pettebone M. (2023). Mechanical stimuli such as shear stress and piezo1 stimulation generate red blood cell extracellular vesicles. Front. Physiol. 14, 1246910. 10.3389/fphys.2023.1246910 37719461 PMC10502313

[B92] Saraiva C. Praca C. Ferreira R. Santos T. Ferreira L. Bernardino L. (2016). Nanoparticle-mediated brain drug delivery: overcoming blood-brain barrier to treat neurodegenerative diseases. J. Control Release 235, 34–47. 10.1016/j.jconrel.2016.05.044 27208862

[B93] Schlesinger M. (2018). Role of platelets and platelet receptors in cancer metastasis. J. Hematol. Oncol. 11 (1), 125. 10.1186/s13045-018-0669-2 30305116 PMC6180572

[B94] Stasi C. Fallani S. Voller F. Silvestri C. (2020). Treatment for COVID-19: an overview. Eur. J. Pharmacol. 889, 173644. 10.1016/j.ejphar.2020.173644 33053381 PMC7548059

[B95] Stoll C. Stadnick H. Kollas O. Holovati J. L. Glasmacher B. Acker J. P. (2011). Liposomes alter thermal phase behavior and composition of red blood cell membranes. Biochim. Biophys. Acta 1808 (1), 474–481. 10.1016/j.bbamem.2010.09.012 20883663

[B96] Sun X. Han X. Xu L. Gao M. Xu J. Yang R. (2017). Surface-Engineering of red blood cells as artificial antigen presenting cells promising for cancer immunotherapy. Small 13 (40), 1701864. 10.1002/smll.201701864 28861943

[B97] Sun M. Wei J. Su Y. He Y. Ge L. Shen Y. (2024). Red blood cell-hitchhiking delivery of simvastatin to Relieve acute respiratory distress syndrome. Int. J. Nanomedicine 19, 5317–5333. 10.2147/IJN.S460890 38859953 PMC11164090

[B98] Talwar N. Jain N. K. (1992). Erythrocyte based delivery system of primaquine: *in vitro* characterization. J. Microencapsul. 9 (3), 357–364. 10.3109/02652049209021250 1403486

[B99] Tsong T. Y. (1991). Electroporation of cell membranes. Biophys. J. 60 (2), 297–306. 10.1016/s0006-3495(91)82054-9 1912274 PMC1260065

[B100] Tzounakas V. L. Karadimas D. G. Papassideri I. S. Seghatchian J. Antonelou M. H. (2017). Erythrocyte-based drug delivery in Transfusion Medicine: Wandering questions seeking answers. Transfus. Apher. Sci. 56 (4), 626–634. 10.1016/j.transci.2017.07.015 28774826

[B101] Usman W. M. Pham T. C. Kwok Y. Y. Vu L. T. Ma V. Peng B. (2018). Efficient RNA drug delivery using red blood cell extracellular vesicles. Nat. Commun. 9 (1), 2359. 10.1038/s41467-018-04791-8 29907766 PMC6004015

[B102] Valkov N. Das A. Tucker N. R. Li G. Salvador A. M. Chaffin M. D. (2021). SnRNA sequencing defines signaling by RBC-derived extracellular vesicles in the murine heart. Life Sci. Alliance 4 (12), e202101048. 10.26508/lsa.202101048 34663679 PMC8548207

[B103] Velliquette R. W. Aeschlimann J. Kirkegaard J. Shakarian G. Lomas-Francis C. Westhoff C. M. (2019). Monoclonal anti-CD47 interference in red cell and platelet testing. Transfusion 59 (2), 730–737. 10.1111/trf.15033 30516833

[B104] Villa C. H. Seghatchian J. Muzykantov V. (2016). Drug delivery by erythrocytes: Primum non nocere. Transfus. Apher. Sci. 55 (3), 275–280. 10.1016/j.transci.2016.10.017 27856317 PMC5424546

[B105] Wannez A. Devalet B. Chatelain B. Chatelain C. Dogne J. M. Mullier F. (2019). Extracellular vesicles in red blood cell concentrates: an overview. Transfus. Med. Rev. 33 (2), 125–130. 10.1016/j.tmrv.2019.02.002 30910256

[B106] Wibroe P. P. Anselmo A. C. Nilsson P. H. Sarode A. Gupta V. Urbanics R. (2017). Bypassing adverse injection reactions to nanoparticles through shape modification and attachment to erythrocytes. Nat. Nanotechnol. 12 (6), 589–594. 10.1038/nnano.2017.47 28396605

[B107] Wiewiora M. Piecuch J. Sedek L. Mazur B. Sosada K. (2017). The effects of obesity on CD47 expression in erythrocytes. Cytom. B Clin. Cytom. 92 (6), 485–491. 10.1002/cyto.b.21232 25914268

[B108] Wu S. Xu W. Shan X. Sun L. Liu S. Sun X. (2025). Targeting splenic myeloid cells with Nanobiologics to Prevent Postablative Pancreatic cancer recurrence via inducing antitumor Peripheral Trained immunity. Adv. Sci. (Weinh) 12 (21), 2413562. 10.1002/advs.202413562 40289661 PMC12140294

[B109] Wynn T. A. Vannella K. M. (2016). Macrophages in tissue Repair, Regeneration, and fibrosis. Immunity 44 (3), 450–462. 10.1016/j.immuni.2016.02.015 26982353 PMC4794754

[B110] Xia Q. Zhang Y. Li Z. Hou X. Feng N. (2019). Red blood cell membrane-camouflaged nanoparticles: a novel drug delivery system for antitumor application. Acta Pharm. Sin. B 9 (4), 675–689. 10.1016/j.apsb.2019.01.011 31384529 PMC6663920

[B111] Xie S. Wu Z. Qi Y. Wu B. Zhu X. (2021). The metastasizing mechanisms of lung cancer: recent advances and therapeutic challenges. Biomed. Pharmacother. 138, 111450. 10.1016/j.biopha.2021.111450 33690088

[B112] Xiong J. Wu M. Chen J. Liu Y. Chen Y. Fan G. (2021). Cancer-erythrocyte hybrid membrane-camouflaged Magnetic nanoparticles with enhanced photothermal-immunotherapy for Ovarian cancer. ACS Nano 15 (12), 19756–19770. 10.1021/acsnano.1c07180 34860006

[B113] Xiu H. Nan X. Guo D. Wang J. Li J. Peng Y. (2022). Gp350-anchored extracellular vesicles: promising vehicles for delivering therapeutic drugs of B cell malignancies. Asian J. Pharm. Sci. 17 (3), 462–474. 10.1016/j.ajps.2022.03.004 35782327 PMC9237600

[B114] Xu Z. Huang J. Zhang T. Xu W. Liao X. Wang Y. (2023). RGD peptide modified RBC membrane functionalized biomimetic nanoparticles for thrombolytic therapy. J. Mater Sci. Mater Med. 34 (4), 18. 10.1007/s10856-023-06719-1 37043085 PMC10097782

[B115] Yang R. Yu Y. (2021). Glucocorticoids are double-edged sword in the treatment of COVID-19 and cancers. Int. J. Biol. Sci. 17 (6), 1530–1537. 10.7150/ijbs.58695 33907516 PMC8071771

[B116] Yang X. Chen M. Weng C. Zhuge D. Jin F. Xiao Y. (2024). Red blood cell membrane-coated nanoparticles enable Incompatible blood transfusions. Adv. Sci. (Weinh) 11 (29), e2310230. 10.1002/advs.202310230 38837643 PMC11304279

[B117] Yu H. Yang Z. Li F. Xu L. Sun Y. (2020). Cell-mediated targeting drugs delivery systems. Drug Deliv. 27 (1), 1425–1437. 10.1080/10717544.2020.1831103 33096949 PMC7594730

[B118] Yu H. Yan J. Li Z. Song T. Ning F. Tan J. (2023). Enhanced photothermal-ferroptosis effects based on RBCm-coated PDA nanoparticles for effective cancer therapy. J. Mater Chem. B 11 (2), 415–429. 10.1039/d2tb02329f 36512437

[B119] Zahednezhad F. Saadat M. Valizadeh H. Zakeri-Milani P. Baradaran B. (2019). Liposome and immune system interplay: challenges and potentials. J. Control Release 305, 194–209. 10.1016/j.jconrel.2019.05.030 31121278

[B120] Zelepukin I. V. Yaremenko A. V. Shipunova V. O. Babenyshev A. V. Balalaeva I. V. Nikitin P. I. (2019). Nanoparticle-based drug delivery via RBC-hitchhiking for the inhibition of lung metastases growth. Nanoscale 11 (4), 1636–1646. 10.1039/c8nr07730d 30644955

[B121] Zhang Y. N. Poon W. Tavares A. J. McGilvray I. D. Chan W. C. W. (2016). Nanoparticle-liver interactions: Cellular uptake and hepatobiliary elimination. J. Control Release 240, 332–348. 10.1016/j.jconrel.2016.01.020 26774224

[B122] Zhang X. Qiu M. Guo P. Lian Y. Xu E. Su J. (2018). Autologous red blood cell delivery of Betamethasone phosphate sodium for long anti-inflammation. Pharmaceutics 10 (4), 286. 10.3390/pharmaceutics10040286 30567356 PMC6320894

[B123] Zhang G. Huang X. Xiu H. Sun Y. Chen J. Cheng G. (2020). Extracellular vesicles: natural liver-accumulating drug delivery vehicles for the treatment of liver diseases. J. Extracell. Vesicles 10 (2), e12030. 10.1002/jev2.12030 33335695 PMC7726052

[B124] Zhao Z. Ukidve A. Krishnan V. Fehnel A. Pan D. C. Gao Y. (2021). Systemic tumour suppression via the preferential accumulation of erythrocyte-anchored chemokine-encapsulating nanoparticles in lung metastases. Nat. Biomed. Eng. 5 (5), 441–454. 10.1038/s41551-020-00644-2 33199847

[B125] Zheng J. Lu C. Ding Y. Zhang J. Tan F. Liu J. (2022). Red blood cell-hitchhiking mediated pulmonary delivery of ivermectin: effects of nanoparticle properties. Int. J. Pharm. 619, 121719. 10.1016/j.ijpharm.2022.121719 35390488 PMC8978457

[B126] Zhu K. Xu Y. Zhong R. Li W. Wang H. Wong Y. S. (2023). Hybrid liposome-erythrocyte drug delivery system for tumor therapy with enhanced targeting and blood circulation. Regen. Biomater. 10, rbad045. 10.1093/rb/rbad045 37250975 PMC10224802

